# QTL Mapping of Agronomic Waterlogging Tolerance Using Recombinant Inbred Lines Derived from Tropical Maize (*Zea mays L*) Germplasm

**DOI:** 10.1371/journal.pone.0124350

**Published:** 2015-04-17

**Authors:** Pervez Haider Zaidi, Zerka Rashid, Madhumal Thayil Vinayan, Gustavo Dias Almeida, Ramesh Kumar Phagna, Raman Babu

**Affiliations:** 1 International Maize and Wheat Improvement Center (CIMMYT)—Asia, C/o International Crop Research Institute for Semi-Arid Tropics (ICRISAT), Patancheru, Hyderabad, India; 2 Directorate of Maize Research (DMR), Pusa Campus, New Delhi, India; 3 Monsanto Company, Uberlândia, Minas Gerais, Brazil; International Rice Research Institute, PHILIPPINES

## Abstract

Waterlogging is an important abiotic stress constraint that causes significant yield losses in maize grown throughout south and south-east Asia due to erratic rainfall patterns. The most economic option to offset the damage caused by waterlogging is to genetically incorporate tolerance in cultivars that are grown widely in the target agro-ecologies. We assessed the genetic variation in a population of recombinant inbred lines (RILs) derived from crossing a waterlogging tolerant line (CAWL-46-3-1) to an elite but sensitive line (CML311-2-1-3) and observed significant range of variation for grain yield (GY) under waterlogging stress along with a number of other secondary traits such as brace roots (BR), chlorophyll content (SPAD), % stem and root lodging (S&RL) among the RILs. Significant positive correlation of GY with BR and SPAD and negative correlation with S&RL indicated the potential use of these secondary traits in selection indices under waterlogged conditions. RILs were genotyped with 331 polymorphic single nucleotide polymorphism (SNP) markers using KASP (Kompetitive Allele Specific PCR) Platform. QTL mapping revealed five QTL on chromosomes 1, 3, 5, 7 and 10, which together explained approximately 30% of phenotypic variance for GY based on evaluation of RIL families under waterlogged conditions, with effects ranging from 520 to 640 kg/ha for individual genomic regions. 13 QTL were identified for various secondary traits associated with waterlogging tolerance, each individually explaining from 3 to 14% of phenotypic variance. Of the 22 candidate genes with known functional domains identified within the physical intervals delimited by the flanking markers of the QTL influencing GY and other secondary traits, six have previously been demonstrated to be associated with anaerobic responses in either maize or other model species. A pair of flanking SNP markers has been identified for each of the QTL and high throughput marker assays were developed to facilitate rapid introgression of waterlogging tolerance in tropical maize breeding programs.

## Introduction

Rainfed maize crop grown during the summer-rainy season, occupying over 80% of the total maize area in the Asian tropics, occasionally face extreme weather conditions that limit crop establishment and yield potential. Moisture availability is seldom optimal for rainfed maize in this region. Inadequate distribution, especially in higher-rainfall areas of Eastern India and many parts of Southeast Asia, causes transient/intermittent water-logging, which is one of the most important constraints for maize production in Asian tropics. Over 18% of the total maize production area in South and Southeast Asia are affected by temporary floods and waterlogging, causing annual production losses of 25–30% [1]. The increasing demand for maize in Asian region is rapidly transforming cropping systems in certain parts of Asia from rice monoculture to more profitable rice-maize systems [2], which currently occupies approximately 3.5M ha area in Asia [3]. However, maize production in this emerging rice-maize cropping sequence often faces the problem of early stage excessive soil moisture, as soils of paddy fields are frequently saturated due to late rains.

Being a non-wetland crop species, maize is highly susceptible to anaerobic soil conditions during germination and early growth stages [4, 5]. However, the extent of damage due to waterlogging stress varies significantly with developmental stage of the crop, and past studies have shown that the maize crop is comparatively more susceptible at germination and early seedling to tasseling stage [6]. Seeds with carbohydrate reserves, including maize seeds, are generally more tolerant of hypoxia (limited oxygen) or even anoxia (no oxygen) than seeds with fatty acid reserves [7, 8]. Therefore, maize seed can germinate under wet soil conditions in the presence of nominal amounts of oxygen [9], but further growth is crippled, if subjected to excess soil moisture stress. At later growth stages some genotypes, with an inbuilt ability to produce adventitious roots at above-ground nodes and to form aerenchyma in the cortical region of adventitious roots have been shown to tolerate excessive moisture to certain extent [10, 11].

Considerable genetic variability has been observed in maize for tolerance to excessive moisture stress [4, 10, 12, 13, 14], which could be favorably exploited in developing maize varieties that can tolerate such hypoxia/anoxia conditions. As described by Mano and Omori [15], three primary physiological mechanisms conditioning water logging tolerance are 1) the ability to grow adventitious/brace roots at the soil surface during flooding conditions; 2) the capacity to form root aerenchyma and (3) tolerance to toxins (e.g., Fe^2+^, H_2_S) under reduced soil conditions. Understanding the genetics of such physiological traits will certainly aid in more precise manipulation of waterlogging tolerance in the maize breeding programs. Waterlogging tolerance is predominantly a polygenic trait in many crop species including maize. Both additive and non-additive effects are important in the inheritance of flooding tolerance in maize and transgressive segregation has been observed for grain yield in F_2_ population involving waterlogging tolerant and sensitive genotypes, thereby indicating the possibility of favorable alleles coming from both the parental lines [16].

Several subspecies (ssp.) of teosinte germplasm have been used as source of flooding tolerance in maize [16] and a number of genetic studies identified genomic regions for several root-related traits under flooding and drained conditions. Mano *et al*. [17] mapped QTL for adventitious root formation under waterlogged conditions on chromosome 4 and 8, using a maize by teosinte (*Zea mays* ssp. *huehuetenangensis*) cross, in which the favorable alleles were contributed by teosinte. Exploring the capacity to form aerenchyma in the cortex of adventitious roots, Mano *et al*. [18] identified three QTL on chromosomes 1, 5 and 8 that together explained close to 45% of phenotypic variance for aerenchyma under non-flooding, drained water conditions in a population derived from B64 x teosinte (*Zea mays* ssp. *nicaraguensis*). A similar study involving another ssp. of teosinte (*Zea mays luxurians*), idenitified different set of QTL for constitutive aerenchyma formation thereby indicating the possibility of pyramiding multiple genomic regions from the different ssp. of teosinte into cultivated maize [19]. Recently, introgression lines tolerant to flooded conditions were developed by transferring genomic regions from Z. mays ssp *nicaraguensis* into maize inbred line (Mi29), which demonstrated the effect of a specific chromosomal segment on the long arm of chromosome 4 donated by teosinte [20]. Construction of high density linkage map in maize by teosinte (*Z*. *mays ssp nicaraguensis*) aided in identification of another QTL on chromosome 1 (Qaer1.06) that was found to regulate constitutive aerenchyma formation [21, 22].

Besides teosinte germplasm, flooding tolerance has also been frequently observed and subsequently mapped on several instances in cultivated maize (*Zea mays ssp mays*). In a study involving a maize F_2_ population, Mano *et al*. [23] detected QTL for the ability to form adventitious roots on soil surface on chromosomes 3, 7 and 8, in which the QTL on chromosome 8 was common with that of teosinte germplasm. A QTL of moderate effect size was reported by Mano *et al*. [24] on bin 3 and 4 of chromosome 1 (1.03/1.04), that explained around 14 and 10% of phenotypic variance for leaf injury and dry weight production respectively under flooded conditions. Flooding tolerance has also been a major focus in many Brazilian maize breeding programs and Anjos e Silva *et al*. [25] identified three loci corresponding to glutamine synthetase (on chromosome 5), zein (on chromosome 4) and triosephosphate isomerase (on chromosome 3) which together explained up to 30% of phenotypic variance for shoot and root dry matter under flooded conditions. A recent genome-wide association study in Chinese germplasm identified four interesting signals on chromosome 5, 6 and 9, each explaining around 15% of phenotypic variance for root/shoot length and fresh weight under waterlogged conditions [26].

From the above-mentioned genetic studies, it is apparent that waterlogging tolerance does exist in elite maize germplasm and the inheritance is likely governed by several QTL. As the heritability of GY and other associated traits under flooded conditions generally tend to be low, the evaluation of tolerance can be easily influenced by environmental conditions, hence the use of marker–assisted selection (MAS) would likely be very effective strategy in these breeding programs [27]. However, the effectiveness of MAS relies on the precise localization of the QTL using the representative breeding germplasm and identification of tightly linked, easy-to use molecular markers. Objectives of the present investigation were to 1) obtain heritability estimates for GY and other secondary traits associated with waterlogging tolerance; 2) determine the extent of association between GY and secondary traits under waterlogged conditions; 3) identify genomic regions influencing waterlogging stress related traits and estimate their effect sizes; 4) determine the degree of overlap between QTL identified from *per se* and TC (test cross) evaluations for waterlogging stress related traits and 5) propose a set of SNP markers and easy to use genotyping assays that physically delimit the identified QTL to enable integrating marker assisted selection for waterlogging tolerance in tropical maize improvement programs.

## Materials and Methods

### Germplasm

The two parental lines used in the study were contrasting for their responses to waterlogging stress. CML311-2-1-3 is an elite but highly sensitive line, whereas CAWL-46-3-1 was tolerant for waterlogging stress. These two parental lines were selected on the basis of their consistent responses in line evaluation trials conducted during 1998–2003 during rainy season at Directorate of Maize Research (DMR), New Delhi, India (28.48N, 77.18E, 228.2 masl). CML-311-2-1-3 was derived from selected segregants in the CIMMYT inbred line CML-311, which was derived from a synthetic population, S89500. CAWL-46-3-1 was derived from a local population (Jaunpur Local) tolerant to vegetative stage waterlogging stress.

The segregating population involving these two parental lines was developed during the dry season of 2005/2006 at DMR Maize Research Station in Hyderabad, India. The population was advanced to F_3_ generation, and thereafter a single-seed descent approach was followed for developing the RILs. A total of 211 lines were developed. In the dry season of 2009/2010, the RILs (S6 stage of inbreeding) were test-crossed with CML451, and the F_1_ seeds (RILs-TC) were harvested for further evaluations. CML451 is a late maturing yellow line, which is used as tester in the tropical maize breeding program of CIMMYT. The RILs and test crosses (TCs) were evaluated under managed waterlogging stress conditions during the rainy season of 2009 at experimental farm of International Crops Research Institute for the Semi-Arid Tropics (ICRISAT), Hyderabad, India (17°53’ N latitude,78°27’ E longitude and 545 masl).

### Managed stress phenotyping

Soil of the experiment field was vertisol with a pH of 7.5. Planting was done in dry soil condition on prepared ridges in field using an alpha lattice design [28] with two replications. All the entries were over sown and thinned to one plant per hill at the V2 growth stage to give a population density of 66,000 plants per hectare. Each entry was planted in one row, 3.0 m long, with 0.20 m spacing within and 0.75 m between rows. Before planting, 60 kg nitrogen (N) per hectare in the form of urea, 60 kg phosphorous per hectare as single super phosphate, 40 kg potassium per hectare as muriate of potash and 10 kg zinc as zinc sulfate were applied as a basal dressing. Second and third doses of N (each 30 kg N per hectare) were side-dressed at knee-high and tasseling stages. Weeds were controlled with pre-emergence application of pendimethalin and atrazine (both at 0.75 kg per hectare) Experiments were kept free from insect, weeds and diseases using recommended post-emergence chemical measures, and managed under optimal agronomic practices. Water logging treatment was applied through flooding at the knee-high stage (V7-8 growth stage) continuously for seven days. At the beginning of the treatment, plots were completely filled with water, and maintained thereafter for 7 days at a depth of 10±0.5cm by supplying water at a rate that exceeded infiltration and evaporation. After completion of the stress treatment the field was completely drained-out.

The TC progenies were screened for water-logging tolerance in a waterlogging-pit facility. The size of pits for this study is 1096cm x 396cm x 43cm that can accommodate 300 pots of the size 30 x 30 cm and volume 14930 cm^3^. Each pot was filled with a mixture of sieved vertisol, alfisol type of soils and farm-yard manure (FYM), in the ratio of 2:2:1. Each entry was planted with two seeds in each pot with five replications using completely randomized design (CRD) following recommended crop management practices. At V2 growth stage, thinning was done and one plant per pot was maintained for further study. At V7-8 stage, the pits were filled with water in a way that water rise above the pot surface at least by 5.0 cm. Water level lost by evaporation was replenished by filling the pits every day to maintain the same water level for 7 days. After 7 days, water level in the pits was reduced to 3–4 cm depth in order to maintain high moisture condition for another 48 hours, following which pits were completely drained-out and plants were kept for recovery and data recording.

### Data observations

Plant height was measured at male flowering, as the average distance from the base to the node bearing the flag leaf in the plot. Leaf senescence was scored at one week after release of the stress, using a 1–10 scale (1 = 10% and 10 = 100% dead leaf area). The *in vivo* chlorophyll concentration of the uppermost fully expanded leaf was determined on all plants per plot/pot at the same time as the senescence score, using a Minolta SPAD-502 chlorophyll meter. Results were averaged to give single plot values. Plant lodging was measured a week after imposing the stress, and measured as percentage of the total number of lodged plants to the total number of plants. Similarly, plant mortality (%) was calculated by counting the number of dead plants per entry, one week after completion of water-logging stress and dividing by the total number of plants. Data on brace root was recorded at 50% male flowering by counting the number of above-ground nodes bearing brace roots. Days from planting to anthesis and silking, indicated by when 50% of plants had one extruded anther or produced one visible silk, were recorded by daily visual observations during the flowering period. Anthesis-silking interval (ASI) was calculated as the difference between number of days to 50% silking and 50% anthesis. Under stress, some of the highly susceptible lines failed to reach 50% silking, resulting in barren plants. In such cases, the maximum days to 50% silking of the trial was considered as days to 50% silking of those genotypes for calculation of ASI. At maturity, ears were harvested and dried to a constant moisture level and grain yield was recorded on a shelled grain basis and adjusted to 15% grain moisture.

### Analysis of phenotypic data

The data from each trait observed were analyzed with Proc Mixed in SAS (9.2) [29]. Blocks were treated as random and entry as fixed effect to obtain the best linear unbiased estimates of the mean from the RIL data set. The mean of each RIL test cross and the best linear unbiased estimates from RILs were then used for QTL analysis.

### Linkage and QTL mapping

Genomic DNA was extracted from young leaves collected in a bulk of 10 plants per RIL entry and according to CIMMYT’s laboratory protocols [30]. The parental lines were genotyped with a set of 1250 SNP markers, for which KASP assays [31] were designed at LGC genomics (formerly Kbiosciences) facility in London, UK. These 1250 SNPs were a subset of 1536 SNPs from Yan *et al*. [32]. Genotyping of the RILs were carried out with 331 polymorphic SNP markers using the KASP platform. Linkage map was constructed using QTL IciMapping ver3.2 software (http://www.isbreeding.net) using the twin criterion of more than 3.0 LOD and a maximum distance of 37.2 cM between two loci [33]. Recombination frequencies between linked loci were transformed into cM distances using Kosambi’s mapping function [34]. The QTL were identified for the adjusted means for each trait using Inclusive Composite Interval Mapping (ICIM) [33] as implemented in QTL IciMapping v.3.2. The walking step in QTL scanning was 1 cM and a likelihood odds (LOD) threshold of 2.5 based on 1000 permutations was chosen for declaring potentially significant QTL for secondary traits associated with drought tolerance [35,36]. The sign of the additive effects of each QTL was used to identify the origin of the favorable alleles in accordance with Lubberstedt *et al*. [37]. Alleles coming from CML311-2-1-3 (waterlogging sensitive) were coded as “2” and from CAWL-46-3-1 (waterlogging tolerant) as “0”. Additive effect was calculated by deducting the phenotypic average of the individuals carrying “0” allele from that of individuals carrying “2” allele. Hence, negative sign of the additive effect of QTL for Grain Yield, Brace Root and Chlorophyll and positive sign for root lodging (%), stem lodging (%), plant mortality (%) and ASI indicates that the favorable allele for the respective traits originated from the waterlogging tolerant line and vice-versa. The proportion of observed phenotypic variation explained (PVE) due to a particular QTL was estimated by the coefficient of determination (R2) from the corresponding linear model (single marker) analysis, and using the maximum likelihood estimates [38]. For each marker that was closest to a QTL, the RILs were grouped into two classes belonging to the two parental alleles of this marker locus. The trait means of two allele classes were compared using a t-test both for significance and for identification of the parent to which the allele having a positive effect on the target trait belonged. A pair of flanking markers that fall in the confidence interval for each of the QTL detected was provided to facilitate marker-assisted selection of the target QTL. Confidence interval was empirically determined by identifying positions on the right and left side of each detected QTL that decay by one LOD unit from that of the QTL and used to identify overlap intervals.

## Results

### Mean performance

Analysis of variance (ANOVA) revealed that mean squares and variance components for genotypes were significant at P < = 0.01 (F-test) for all the observed traits evaluated under waterlogging stress in both *per se* (Table 1) as well as TC experiments. Except ASI, most of the traits exhibited moderate to high heritability estimates indicating the repeatable nature of the traits under waterlogged conditions. Among the various pairs of correlations generated, significant positive associations between brace roots and chlorophyll content with GY and significant negative associations between root and stem lodging with GY were most notable, suggesting their importance in indirect selection for water logging tolerance (S1 Table).

**Table 1:** Mean, variance and heritability estimates for parental lines and RIL families based on evaluation under waterlogging conditions.

Trait	Mean±SE	P1 (WLT)	P2 (WST)	Range in RIL	H
Grain yield (t/Ha)	0.80±0.02	1.8	0.4	0.06–2.22	0.57
ASI (Days)	6.62±0.23	1.0	7.8	-3.28–29.79	0.27
Ears per plant (Nos)	1.66±0.05	2.6	1.2	0.00–4.29	0.47
Plant height (cm)	103.69±11.07	138.0	126.3	58.61–160.44	0.46
Ear height (cm)	32.20±0.56	52.4	48.6	13.84–67.86	0.45
Brace roots (No)	1.66±0.05	3.5	0.6	0.00–4.29	0.78
Ear position (Ratio)	0.31±0.00	0.32	0.28	0.16–0.54	0.44
Chlorophyll (SPAD)	16.99±0.28	22.32	17.64	7.50–29.60	0.67
Root lodging (%)	14.6±1.7	4.2	47.8	0.00–76.26	0.91
Stem lodging (%)	3.9±0.50	1.5	35.6	0.00–51.20	0.87

WLT—Waterlogging tolerant parent (CML311-2-1-3); WST—Waterlogging sensitive parent (CAWL-46-3-1); H—Heritability (Broad sense) estimate.

### Linkage mapping

All the ten maize chromosomes were represented in the linkage map constructed with ten linkage groups (Corresponding to ten chromosomes), spanning a total length of 2,008.2 cM at an average marker interval of 12.4 cM. Almost all bin locations in the maize genome were represented in this linkage map except for some such as 1.06, 2.04, 7.02 and 8.04 due to lack of polymorphic loci obtained from these regions. Wherever discrepancies were observed with respect to order and position of markers between linkage and physical distances, forced reordering of markers were carried out according to the physical locations based on AGP v2 of maize reference sequence [39, 40].

Missing data in the genotyping of mapping population across 331 SNP loci were below 5%. Chi-square tests showed that 68 markers deviated from the expected ratio of 1:1 and were subsequently removed for the QTL analyses. Allele frequencies of CML311 were slightly higher than CAWL-46-3-1. The contribution of alleles from the susceptible parent (CML311) in the individuals of the mapping population was found to be 59.4%, ranging from 49.1 to 65.2%, while that of alleles from the tolerant parent was 41.6%, ranging from 33.2 to 52.7%. The marginal upward bias for the susceptible parent could have been due to smaller sample size, which in turn led to inadvertent genetic drift during the development of RIL families through multiple filial generations.

### QTL for waterlogging tolerance

#### Grain yield (GY)

**Fig 1:**
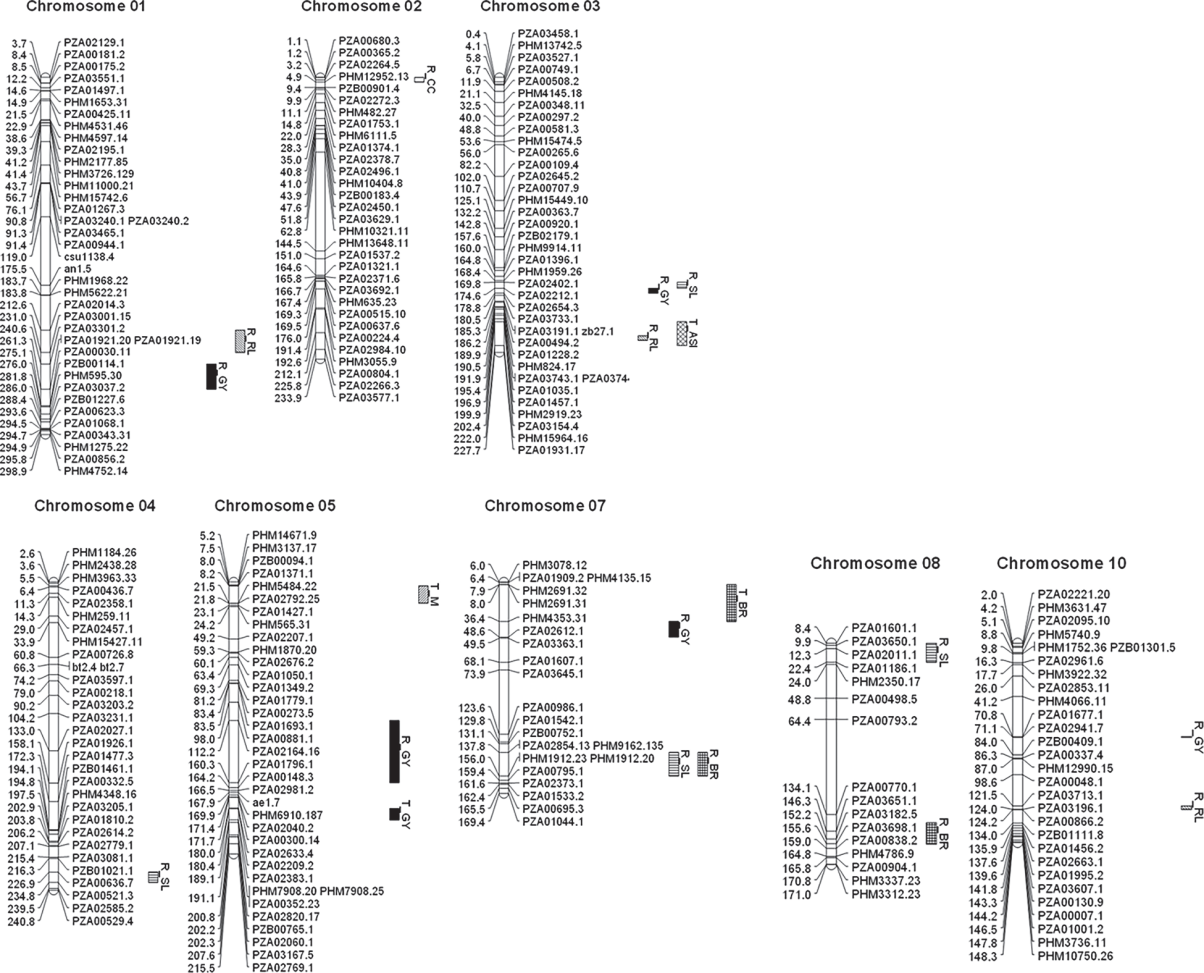
Linkage groups along with QTL identified for traits associated with waterlogging tolerance using RIL and TC phenotypes (R_: Identified using RIL dataset, T_: Identified using TC data set, Traits: GY—Grain yield, RL—Root lodging, SL—Stem lodging, BR—Brace roots, M—Plant Mortality %, CC—Chlorophyll content, ASI—Anthesis-Silking interval).

The RIL evaluations revealed five QTL for GY under waterlogging stress on chromosomes 1, 3, 5, 7 and 10 and one QTL on chromosome 5 using RIL TC dataset (Tables 2 and 3, Fig 1). The position of the QTL identified in the RIL-TC experiment on chromosome 5 was close to that of GY in RIL and accounted for 3.4 and 8% of the phenotypic variance respectively. The trait enhancing alleles in both the cases were contributed by the water logging tolerant parent line. The five QTL detected in RIL experiment together explained close to 30% of the phenotypic variance. The favorable alleles at QTL on chromosomes 1, 3 and 5 were contributed by the waterlogging tolerant parent, while the susceptible parent donated the favorable alleles at the rest of the two loci. The additive effects of the QTL from water logging tolerant parental line were significant and substantial which ranged from 520 to 640 Kg/ha). Interestingly, the favorable allele at QTL on chromosome 7 was contributed by the waterlogging susceptible parent (CML311), which had an additive effect of around 500 Kg/ha.

**Table 2:** QTL identified for waterlogging tolerance using RIL phenotypes.

Trait\Chromosome	Flanking Markers and their physical positions (Mb)	Confidence Interval (Mb)	LOD	R2 (%)	Additive Effect
Grain yield (t/ha)					
1	PZA03301.2 (240.57)—PZA01921.20 (261.31)	219.62–265.11	6.4	5.0	-0.60
3	PZA02212.1 (174.55)—PZA02654.3 (178.77)	157.97–198.52	6.1	4.2	-0.64
5	PZA02164.16 (112.18)—PZA01796.1 (160.32)	103.79–178.62	4.5	8.0	-0.52
7	PHM4353.31 (36.39)—PZA02612.1 (48.61)	6.26–61.41	11.6	6.1	0.49
10	PZA01677.1 (70.80)—PZA02941.7 (71.12)	40.93–83.74	5.4	3.6	0.11
Root lodging (%)					
1	PZA02014.3 (212.56)—PZA03001.15 (231.04)	212.56–250.98	3.3	3.8	12.39
3	PZA03458.1 (205.96)—PHM13742.5 (215.92)	192.95–220.56	10.4	6.3	17.67
10	PZA03713.1 (121.49)—PZA03196.1 (124.01)	106.34–124.01	5.3	6.1	16.22
Stem lodging (%)					
3	PZA02402.1 (169.77)—PZA02212.1 (174.55)	168.04–182.70	5.2	8.6	14.64
4	PZA00636.7 (226.88)—PZA00521.3 (234.78)	220.33–246.32	13.0	9.2	12.90
7	PHM9162.135 (137.83)—PHM1912.23 (155.97)	113.74–155.97	12.8	6.4	17.65
8	PZA01601.1 (8.40)—PZA01186.1 (22.42)	8.40–37.05	12.7	7.1	15.04
Brace Root (No.)					
7	PHM9162.135 (137.83)—PHM1912.23 (155.97)	113.74–155.97	14.4	4.8	-4.49
8	PZA03698.1 (155.62)—PZA00838.2 (159.04)	148.54–162.81	11.9	4.2	-4.81
Chlorophyll (SPAD)					
2	PZA00365.2 (1.22)—PHM12952.13 (4.88)	1.11–9.44	3.1	13.6	1.6

**Table 3:** QTL identified for waterlogging tolerance using RIL test cross phenotypes.

Trait\Chromosome	Flanking Markers and their physical positions (Mb)	Confidence interval (Mb)	LOD	R^2^ (%)	Additive Effect
Grain yield (t/ha)					
5	PZA02383.1 (180.42)—PZA02209.2 (189.14)	171.7–191.1	2.8	3.4	-0.01
Anthesis Silking Interval (days)					
3	PZA03154.4 (203.31)—PHM15964.16 (213.61)	191.9–222.0	4.8	6.8	2.08
Brace Root (No.)					
7	PHM2691.31 (7.94)—PHM4353.31 (36.39)	6.0–48.6	3.1	2.6	0.79
Plant Mortality (%)					
5	PZA01371.1 (8.22)—PHM5484.22 (21.50)	7.5–23.1	2.8	12.3	12.25

#### Secondary traits associated with waterlogging tolerance

A total of 13 QTL were detected across all the chromosomes for the 10 waterlogging component traits using RIL and three traits using TC dataset (Tables 2 and 3, Fig 1). The detected QTL individually accounted for 3–13.6% of the phenotypic variance. Two QTL were detected on chromosomes 7 and 8 for brace root trait, favorable alleles for which were contributed by the tolerant parent. A QTL on chromosome 7 was detected for brace root in RIL-TC experiment, which however differed considerably in terms of physical location from that of RIL evaluations. It was interesting to note that the physical location of brace root QTL identified on chromosome 7 in TC experiment overlapped with the GY QTL detected based on RIL evaluations. A relatively large effect QTL was identified on chromosome 2 for chlorophyll content, which explained close to 14% of phenotypic variance and the favorable allele was contributed by the waterlogging susceptible but elite parent (CML311). Seven QTL with moderate additive effects were detected for root and stem lodging percentage under waterlogged conditions especially in *per se* evaluations for which the favorable alleles were entirely contributed by the waterlogging tolerant parent (Table 2). Another relatively large effect QTL was notable on chromosome 5 for plant mortality percentage in TC evaluations, which is an indication of early stage seedling tolerance to waterlogged conditions in contrast to stem and root lodging percent that correspond to later stages of adult plant tolerance. For ASI, a QTL on chromosome 3 accounted for around 7% of phenotypic variance in TC evaluations. As expected, at both these loci for plant mortality percentage and ASI, the favorable alleles (‘trait-reducing’) were contributed by the waterlogging tolerant parent.

#### Candidate genes underlying QTL intervals

Based on the ‘Named Genes’ annotation track (http://www.plantgdb.org/ZmGDB), 22 candidate genes with known functions were identified within the physical intervals delimited by the confidence interval of the QTL influencing GY and other secondary traits associated with waterlogging tolerance, of which six of them have previously been demonstrated to be associated with anaerobic responses in either maize or other model species like *Arabidopsis* and Tobacco (Table 4). The range of putative functions varied from conditioning metabolic responses such as biosynthesis of lipophilic compounds to early development of brace roots under flooded conditions. The delimited interval on chromosome 3 for ASI harbored a MADS domain transcription factor (zmm16), which has been implicated in reproductive organ development.

**Table 4:** Putative candidate genes identified in the physical intervals delimited by the flanking markers of the QTL influencing GY and secondary traits under waterlogged conditions.

chr	Interval (Mb)	Putative candidate genes	Gene Id	Functions	References
1	240–261	Cytochrome P-450- 8(cyp8)	GRMZM2G167986	Biosynthesis of endogenous lipophilic compounds upon hypoxia	[59]
		TATA-binding protein	GRMZM2G149238	Anaerobic gene expression	[60,61]
3	174–178	phosphoinositide dependent protein kinase 1	GRMZM2G097821	Anaerobic signal transduction	[62]
3	203–213	MADS domain transcription factor (zmm16)	GRMZM2G110153	Reproductive organ development	[47]
5	8–21.5	Cytochrome b6	GRMZM2G463640	Selective activation under hypoxic conditions	[48]
		Single myb histone 6	GRMZM2G095239	Regulation of alcohol dehydrogenase under low oxygen conditions	[51]
5	112–160	Cysteine Protease (ccp1)	GRMZM2G098298	Anoxia-induced root-tip death	[42]
7	137–155	Glutathione S transferase16	GRMZM5G895383	Metabolic processes relating to early development of brace roots	[44]

## Discussion

Agronomically, waterlogging tolerance is the maintenance of relatively high grain yields under waterlogged as compared to non-waterlogged conditions. Physiological mechanisms controlling such tolerance vary from whole plant regulation to intracellular signaling and programmed cell death. Identification of key donor lines conferring consistent waterlogging tolerance and unravelling genetic factors that influence vital physiological regulators under anaerobic/hypoxic conditions will enable not only gaining better understanding of critical mechanisms but also efficient incorporation of waterlogging tolerance in the maize breeding pipeline.

The previous genetic investigations on waterlogging tolerance revealed that the trait is likely polygenic in maize and affected by several mechanisms and complicated by confounding factors such as temperature, plant development stage, nutrient availability, soil type and sub-soil topography [27]. Direct selection on GY under waterlogged conditions is the most favored strategy by the breeders, which however suffers from low heritability estimates for the reasons stated above. Secondary traits such as anthesis-silking Interval (ASI), number of nodes with brace roots (BR), root and shoot biomass, chlorophyll content, plant and ear height, etc. have the potential to be used as indirect selection indices along with grain yield for waterlogging tolerance in maize. Here, we have presented optimized trait measurement approaches especially for such secondary traits that yielded moderate to high heritability (h^2^) estimates. Some of the traits like BR and stem and root lodging percent not only recorded high h^2^ estimates but also robust favorable correlations with GY, thereby warranting their inclusion in the selection strategy for enhanced waterlogging tolerance. Similar strategies of using adventitious root and aerenchyma formation, leaf injury, dry matter production as indicators of waterlogging tolerance have been reported in maize [17,23,24,41].

In the current study, we have identified additive QTL with relatively moderate effects for grain yield under waterlogged conditions, for which favorable alleles were contributed by the water logging tolerant parent on chromosomes 1, 3 and 5. It is interesting to note that QTL for root lodging and ASI were mapped on the overlapping intervals with that of GY QTL on chromosomes 1 and 3. Investigating the ability to form root aerenchyma formation in a cross involving *Zea nicaraguensis*, Mano *et al*. [18] identified two QTL in the overlapping physical interval of GY QTL identified in the current study on chromosome 1 and 5. Similarly, a mapping study on the ability to form adventitious roots identified a QTL on chromosome 3, which co-located with the GY QTL in the present study. Contrary to the above-mentioned three QTL regions, favorable alleles at the QTL on chromosome 7 and 10 were contributed by the elite but waterlogging sensitive parental line. In the absence of optimal performance data, it will however be difficult to point out whether the identified QTL in these locations actually correspond to waterlogging tolerance or general GY performance. Nevertheless, identification of such genomic regions along with pairs of flanking markers will facilitate marker-aided selection of superior transgressive segregants in the early filial generations of breeding programs targeted towards developing improved maize germplasm that perform equally well under optimal and waterlogged conditions. Recently, Zhang *et al*. [26] carried out a genome-wide scan of seedling waterlogging tolerance in a diverse collection of Chinese maize germplasm which revealed an interesting region on chromosome 5.04 (~ 159–165 Mb as indicated by PZE-105105668) that explained 15 to 20% of phenotypic variance for root/shoot fresh & dry weights under waterlogged conditions and overlapped with the QTL identified for GY in the current study. Zhang *et al*. [26] further validated this region on 5.04 in a population that was backcross-derived from a cross involving waterlogging tolerant (HZ32) and sensitive (K12) lines, thereby indicating the potential of this region to be further explored in marker assisted introgressions. Analysis of candidate genes in the delimited interval of 112–160 Mb on chromosome 5 revealed a cysteine protease gene (ccp1), which has been previously demonstrated to be associated with anoxia-induced root tip death in maize [42].

Two QTL regions were identified on chromosomes 7 and 8 for brace root development which had significant positive association with GY under waterlogged conditions (Tables 2 and 3). The favorable alleles at both the QTL were contributed by waterlogging tolerant parent and together explained close to 10% of phenotypic variance. Independently, for root lodging, three QTL were detected on chromosomes 1, 3 and 10 at which the favorable allele were contributed by waterlogging tolerant parent (Table 2). Based on the evaluation of immortalized F_2_ populations derived from recombinant inbred lines of CML288 (tropical) and Huanzao4 (temperate Chinese), Ku *et al*. [43] reported QTL for total and effective brace root tier number at the same physical interval (~8–15 Mb) on chromosome 8, where QTL for brace root were identified in the current study. Root and stem lodging in maize are considered critical attributes for maintaining crop productivity especially under waterlogged conditions. A total of 7 QTL were identified in the present study for root and shoot lodging and the favorable alleles were solely contributed by waterlogging tolerant parent at all the 7 loci. The QTL identified on chromosome 7 for brace root overlapped with that of genomic region responsible for stem lodging (Fig 1). Reference to functional annotation of the delimited genomic interval on chromosome 7 (137–155 Mb) identified a glutathione S-transferase16 (gst16) gene, which is predicted to play a key role in the metabolic processes leading to early stage brace root development [44].

ASI was found to be more relevant in the hybrid (Test cross) trial and a strong QTL on chromosome 3 was identified (Table 3), wherein the favorable allele coming from the waterlogging parent decreased the interval between male and female flowering by ~2 days. Recently, Almeida *et al*. [45,46] performed metaQTL analyses across three tropical populations for GY, ASI and other secondary traits, which identified a hotspot QTL region on chromosome 3 (~170–214 Mb) that contained an important candidate gene, zmm16 (MADS box domain transcription factor), which is associated with reproductive organ development in maize [47]

Waterlogging tolerance especially at the early stages of plant development such as seedling growth phase can provide substantial advantage for subsequent survival under flooded conditions during the later stages of growth. The QTL identified on chromosome 5 (~ 7-23Mb) for plant mortality % based on TC experiment explained more than 10% of phenotypic variance and merits attention for marker-assisted introgressions. This physical interval harbored two important genes related to waterlogging tolerance 1) a cytochrome b6 gene (GRMZM2G463640), which is oxygen-dependent and is known to change sub-unit structure and holoenzyme levels, especially during low oxygen conditions [48]. The Arabidopsis genome contains as many as 286 different cytochrome P450 genes, some of which play crucial roles in the biosynthesis of a variety of endogenous lipophilic compounds and cross talk in the responses to abiotic and biotic stresses [49,50]; 2) single myb histone 6 gene (GRMZM2G095239), whose related family member (*myb2*) has been demonstrated to play a strong regulatory role in the induction of alcohol dehydrogenase (*adh1*) during low oxygen conditions [51].

The partial mismatch between QTL identified based on *per se* and TC phenotypes is likely because of one or combination of below mentioned factors—strong tester effects, which may mask the parental allele performance [52, 53], smaller population size [54, 55], field versus pit phenotyping of *per se* and TC individuals, high QTL x Environment interaction [56] and genetic architecture of the trait (such as epistatic interactions as reported by [52, 57,58]).

Reliable screening methods, consistent sources of tolerance and breeding critical information such as number and effect sizes of various genomic regions influencing tolerance traits will help in enhancing the success of breeding programs. The screening methods reported and set of QTL identified in the current study for GY and various other secondary traits will be helpful in designing marker-aided pyramiding or introgressions in the tropical maize breeding program aimed at efficient incorporation of waterlogging tolerance.

## Supporting Information

S1 Table Genotypic correlation matrix among waterlogging tolerance related traits. (DOCX)

## References

[1] Cairns JE, Sonder K, Zaidi PH, Verhulst N, Mahuku G, Babu R, et al Maize production in a changing climate. Adv Agron. 2012;144: 1–58.

[2] Waddington SR, Elahi NE, Khatun F. The Expansion of Rice-maize Systems in Bangladesh. In:The Symposium on Emerging Rice-Maize Systems in Asia. ASA-CSSA-SSSA, International Annual Meetings, Indianapolis, Indiana, USA. 12–16 November 2006.

[3] Timsina J, Jat ML, Majumdar K. Rice-maize systems of South Asia: current status, future prospects and research priorities for nutrient management Plant Soil. 2010;335(1): 65–82.

[4] Mano Y, Muraki M, Komatsu T, Fujimori M, Akiyama F, Takamizo T. Varietal difference in pre-germination flooding tolerance and waterlogging tolerance at the seedling stage in maize inbred lines. Jpn J Crop Sci. 2002;71(3): 361–367.

[5] Zaidi PH, Rafique S, Rai PK, Singh NN, Srinivasan G. Tolerance to excess moisture in maize (*Zea mays* L.): susceptible crop stages and identification of tolerant genotypes. Field Crop Res. 2004;90(2–3): 189–202.

[6] Zaidi PH, Maniselvan P, Sultana R, Yadav M, Singh RP, Singh SB et al Importance of secondary traits in improvement of maize (*Zea mays* L.) for enhancing tolerance to excessive soil moisture stress. Cereal Res Commun. 2007;35: 1427–1435.

[7] Al-Ani A, Bruzau F, Raymond P, Saint-Ges V, Leblanc JM, Pradet A. Germination, respiration, and adenylate energy charge of seeds at various oxygen partial pressures. Plant Physiol. 1985;79: 885–890. 16664510 10.1104/pp.79.3.885 PMC1074989

[8] Raymond P, Al-Ani A, Pradet A. ATP production by respiration and fermentation, and energy charge during aerobiosis and anaerobiosis in twelve fatty and starchy germinating seeds. Plant Physiol. 1985;79: 9879–9884. 10.1104/pp.79.3.879 PMC1074988 16664509

[9] Van Toai TT, Saglio P, Ricad B, Pradit A. Development regulation of anoxic stress tolerance in maize. Plant Cell Environ. 1995;18: 937–942.

[10] Rathore TR, Warsi MZK, Lothrop JE, Singh NN. Production of maize under excess soil moisture (water-logging) conditions. In: 1st Asian Regional Maize Workshop, PAU (Punjab Agricultural University), Ludhiana; 10–12 Feb 1996. pp. 56–63.

[11] Zaidi PH, Rafique S, Singh NN. Response of maize (*Zea mays* L.) genotypes to excess moisture stress: morpho-physiological effects and basis of tolerance. Eur J Agron. 2003;19: 383–399.

[12] Torbert HA, Hoeft RG, Vanden-Heuvel RM, Mulvaney RL, Hollinger SE. Short-term excess water impact on corn yield and nitrogen recovery. J Prod Agric. 6: 337–344.

[13] Rathore TR, Warsi MZK, Zaidi PH, Singh NN. Waterlogging problem for maize production in Asian region. TAMNET News Letter. 1997;4: 13–14.

[14] Zaidi PH, Rafique S, Singh NN, Srinivasan G. Excess moisture tolerance in maize—progress and challenges. In: Proc. 8th Asian Regional Maize Workshop, Bangkok, Thailand; 5–9 August 2002. pp.398–412.

[15] Mano Y, Omori F.Breeding for flooding tolerant maize using “teosinte” as a germplasm resource. Plant Root. 2007;1: 17–21.

[16] Anjos e Silva SD, Maria J, Claudia FL, Antonio CO, Jose F. Inheritance of tolerance to flooded soils in maize. Crop Breeding and Applied Biotechnology. 2007;7:165–172.

[17] Mano Y, Muraki M, Fujimori M, Takamizo T. Varietal difference and genetic analysis of adventitious root formation at the soil surface during flooding in maize and teosinte seedlings. Jpn J Crop Sci. 2005a;74: 41–46.

[18] Mano Y, Omori F, Takamizo T, Kindiger B, Bird R, Loaisiga CH, et al QTL mapping of root aerenchyma formation in seedlings of a maize × rare teosinte “Z. *nicaraguensis*” cross. Plant Soil. 2007;295: 103–113.

[19] Mano Y, Omori F, Kindiger B, Takahashi H. A linkage map of maize × teosinte Zea *luxurians* and identification of QTLs controlling root aerenchyma formation. Mol Breed. 2008;21: 327–337.

[20] Mano Y, Omori F. Flooding tolerance in interspecific introgression lines containing chromosome segments from teosinte (Zea *nicaraguensis*) in maize (*Zea mays* subsp. mays). Ann Bot. 2013; 10.1093/aob/mct160 PMC3783227 23877074

[21] Mano Y, Omori F. High-density linkage map around the root aerenchyma locus Qaer1.06 in the backcross populations of maize Mi29 × teosinte “Zea *nicaraguensis*”. Breed Sci. 2009;59: 427–433.

[22] Mano Y, Omori F, Loaisiga CH, Bird RM. QTL mapping of above-ground adventitious roots during flooding in maize × teosinte “Zea *nicaraguensis*” backcross population. Plant Root. 2009;3: 3–9.

[23] Mano Y, Muraki M, Fujimori M, Takamizo T, Kindiger B. Identification of QTL controlling adventitious root formation during fiooding conditions in teosinte (*Zea mays* ssp. *huehuetenangensis*) seedlings. Euphytica. 2005b;142: 33–42.

[24] Mano Y, Muraki M, Takamizo T. Identification of QTL controlling flooding tolerance in reducing soil conditions in maize (*Zea mays* L.) seedlings. Plant Prod Sci. 2006;9(2): 176–181.

[25] Sérgio D, Maria J, Cláudia FL, Antonio CO and José F. Genetic parameters and QTL for tolerance to flooded soils in maize. Crop Breeding and Applied Biotechnology. 2005;5: 287–293.

[26] Zhang X, Tang B, Yu F, Li L, Wang M, Xue Y, et al Identification of major QTL for waterlogging tolerance using genome-wide association and linkage mapping of maize seedlings. Plant Mol Biol Rep. 2013;31: 594–606.

[27] Zhou MZ.Improvement of plant waterlogging tolerance In: Mancuso S, Shabala S, eds. Waterlogging signalling and tolerance in plants. Heidelberg, Germany: Springer-Verlag; 2010 pp267–285.

[28] Patterson HD, Williams ER. A new class of resolvable incomplete block designs. Biometrika. 1976;63: 83–92.

[29] SAS Institute Inc., (1987) SAS Campus Drive, Cary, North Carolina 27513.

[30] CIMMYT. The Applied Biotechnology Centre’s Manual of Laboratory Protocol. 1st ed. Mexico D.F., CIMMYT; 2001.

[31] Semagn K, Babu R, Sarah H, Olsen M. Single nucleotide polymorphism genotyping using Kompetitive Allele Specific PCR (KASP): overview of the technology and its application in crop improvement. Mol Breed. 2013;33: 1–14.

[32] Yan JB, Yang XH, Shah T, Sánchez H, Li JS, Warburton ML, et al High-throughput SNP genotyping with the GoldenGate assay in maize. Mol Breed. 2010;25: 441–451.

[33] Li H, Ye G, Wang J. A modified algorithm for the improvement of composite interval mapping. Genetics, 2007;175: 361–374. 17110476 10.1534/genetics.106.066811 PMC1775001

[34] Kosambi DD.The estimation of map distance from recombination values. Annals of Eugenics. 1944;12: 172–175.

[35] Ribaut JM, Monneveux P, Glaszmann JC, Leung H, Van Hintum T, de Vicente C. International programs and the use of modern biotechnologies for crop improvement In:Moore P, Ming R, eds Genomics of Tropical Crop Plants. NY: Springer 2008 pp21–63.

[36] Tuberosa R, Salvi S, Sanguineti MC, Landi P, Maccaferri M, Conti S. Mapping QTL regulating morphophysiological traits and yield: case studies, shortcomings and perspectives in drought-stressed maize. Ann Bot. 2002;89: 941–963. 12102519 10.1093/aob/mcf134 PMC4233811

[37] Lubberstedt T, Melchinger AE, Schon CC, Utz H, Klein D. QTL mapping in testcrosses of European flint lines of maize. I. Comparison of different testers for forage yield traits. Crop Sci. 1997;37: 921–931.

[38] Basten CJ, Weir B, Zeng ZB. QTL Cartographer: A reference manual and tutorial for QTL mapping Department of Statistics, North Carolina State University, Raleigh, NC 1997.

[39] http//www.maizesequence.org. Accessed 2014 May 10

[40] http//www.gramene.org. Accessed 2014 May 08

[41] Bird RMK. A remarkable new teosinte from Nicaragua: Growth and treatment of progeny. Maize Gen Coop Newsl. 2000;74: 58–59.

[42] Subbaiah CC, Kolliparar K, Sachs MM. Potential involvement of Maize AIP in the anoxia-induced death of root tip. In: 39th Annual Maize Genetic Conference, Lake Geneva, WI, 98; 1999.

[43] Ku LX, Sun ZH, Wang CL, Zhang J, Zhao RF, Liu HY, et al QTL mapping and epistasis analysis of brace root traits in maize. Mol Breed.30: 697–708.

[44] Li Y, Fu Y, Huang JG, Wu CA, Cheng CZ. Transcript profiling during the early development of the maize brace root via Solexa sequencing. FEBS Journal. 2010;278: 156–166. 10.1111/j.1742-4658.2010.07941.x 21122072

[45] Almeida GD, Makumbi D, Magorokosho C, Nair S, Bore´m A, Ribaut JM, et al QTL mapping in three tropical maize populations reveals a set of constitutive and adaptive genomic regions for drought tolerance. Theor Appl Genet. 2013;126: 583–600. 10.1007/s00122-012-2003-7 23124431 PMC3579412

[46] Almeida GD, Nair S, Bore´m A, Cairns J, Trachsel S, Ribaut JMet al. Molecular mapping across three populations reveals a QTL hotspot region on chromosome 3 for secondary traits associated with drought tolerance in tropical maize. Mol Breed. 2014;34: 701–715. 25076840 10.1007/s11032-014-0068-5 PMC4092235

[47] Setter TL, Yan J, Warburton M, Ribaut JM, Xu Y, et al Genetic association mapping identifies single nucleotide polymorphisms in genes that affect abscisic acid levels in maize floral tissues during drought. J Exp Bot. 2011;62: 701–716. 10.1093/jxb/erq308 21084430 PMC3003815

[48] Bunn HF, Poyton RO. Oxygen sensing and molecular adaptation to hypoxia. Physiol Rev. 1996;76: 839–885. 8757790 10.1152/physrev.1996.76.3.839

[49] Paquette SM, Bak S, Feyereisen R. Intron-exon organization and phylogeny in a large superfamily, the paralogous cytochrome P450 genes of Arabidopsis thaliana. DNA Cell Biol. 2009;19: 307–317. 10.1089/10445490050021221 10855798

[50] Narusaka Y, Narusaka M, Seki M, Umezawa T, Ishida J, Nakajima M, et al Crosstalk in the responses to abiotic and biotic stresses in Arabidopsis: analysis of gene expression in cytochrome P450 gene superfamily by cDNA microarray. Plant Mol Biol. 2004;55: 327–342. 15604685 10.1007/s11103-004-0685-1

[51] Hoeren FU, Dolferus R, Wu YR, Peacock WJ, Dennis ES. Evidence for a role for AtMYB2 in the induction of the Arabidopsis alcohol dehydrogenase gene (ADH1) by low oxygen. Genetics. 1998;149: 479–490. 9611167 10.1093/genetics/149.2.479 PMC1460183

[52] Peng B, Li Y, Wang Y, Liu Z, Zhang Y, Tan W, et al Correlations and comparisons of quantitative trait loci with family *per se* and testcross performance for grain yield and related traits in maize. Theor Appl Genet. 2013;126: 773–789. 10.1007/s00122-012-2017-1 23183923

[53] Mei HW, Luo LJ, Ying CS, Wang YP, Yu XQ, Guo LB, et al Gene actions of QTLs affecting several agronomic traits resolved in a recombinant inbred rice population and two testcross populations. Theor Appl Genet. 2003;107: 89–101. 12721635 10.1007/s00122-003-1192-5

[54] Carlborg O, Haley CS. Epistasis: too often neglected in complex trait studies? Nat Rev Genet. 2004;5: 618–625. 15266344 10.1038/nrg1407

[55] Utz HF, Melchinger AE, Schon CC. Bias and sampling error of the estimated proportion of genotypic variance explained by quantitative trait loci determined from experimental data in maize using cross validation and validation with independent samples. Genetics. 2000;154:1839–1849. 10866652 PMC1461020

[56] Holland JB. Genetic architecture of complex traits in plants. Curr Opin Plant Biol. 2007;10: 156–161. 17291822 10.1016/j.pbi.2007.01.003

[57] Mihaljevic R.Biometrical analyses of epistasis and the relationship between line *per se* and testcross performance of agronomic traits in elite populations of European maize (*Zea mays* L.). Dissertation, University of Hohenheim, Germany. 2005.

[58] Melchinger AE, Utz HF, Schon CC. Quantitative trait locus (QTL) mapping using different testers and independent population samples in maize reveals low power of QTL detection and large bias in estimates of QTL effects. Genetics. 1998;149: 383–403. 9584111 10.1093/genetics/149.1.383 PMC1460144

[59] Yang CY, Hsu FC, Li JP, Wang NN, Shih MC. The AP2/ERF transcription factor AtERF73/HRE1 modulates ethylene responses during hypoxia in Arabidopsis. Plant Physiol. 2011;156: 202–212. 10.1104/pp.111.172486 21398256 PMC3091062

[60] Geffers R, Sell S, Cerff R, Hehl R. The TATA box and a Myb binding site are essential for anaerobic expression of a maize GapC4 minimal promoter in tobacco. Biochimica and Biophysica Acta. 2001;1521: 120–125. 11690643 10.1016/s0167-4781(01)00302-5

[61] Köhler U, Liaud MF, Mendel RR, Cerff R, Hehl R. The maize GapC4 promoter confers anaerobic reporter gene expression and shows homology to the maize anthocyanin regulatory locus C1. Plant Mol Biol. 1995;29: 1293–1298. 8616225 10.1007/BF00020469

[62] Reggiani R, Laoreti P. Evidence for the involvement of phospholipase C in the anaerobic signal transduction. Plant Cell Physiol. 2000;41: 1392–1396. 11134425 10.1093/pcp/pcd073

