# Supplementary material for: QTL Mapping of Agronomic Waterlogging Tolerance Using Recombinant Inbred Lines Derived from Tropical Maize (Zea mays L) Germplasm
Source: PLoS One. 2015 Apr 17;10(4):e0124350. doi: 10.1371/journal.pone.0124350 (PMC4401703; doi:10.1371/journal.pone.0124350)
Supplement: S1 Table — (DOCX) [file pone.0124350.s001.docx]

**Table S1. Genotypic correlation matrix among waterlogging tolerance related traits based on the RIL evaluations**

| **ASI** |  |  |  |  |  |  |  |  |  |  |
| --- | --- | --- | --- | --- | --- | --- | --- | --- | --- | --- |
| **BR** | -0.08 |  |  |  |  |  |  |  |  |  |
| **CC** | -0.17* | 0.04 |  |  |  |  |  |  |  |  |
| **EPP** | -0.04 | 0.02 | 0.15* |  |  |  |  |  |  |  |
| **EH** | -0.20* | 0.12* | 0.14* | 0.02 |  |  |  |  |  |  |
| **EP** | -0.14* | 0.10* | 0.19* | 0.00 | 0.71** |  |  |  |  |  |
| **GY** | -0.19* | 0.38** | 0.43** | 0.19* | 0.14* | 0.07 |  |  |  |  |
| **RL** | 0.01 | -0.37** | -0.10* | 0.02 | 0.05 | 0.00 | -0.36** |  |  |  |
| **SL** | 0.11* | -0.19* | -0.16* | -0.10* | 0.01 | -0.04 | -0.32** | 0.09 |  |  |
| **PH** | -0.15* | 0.06 | 0.01 | -0.02 | 0.62** | -0.01 | 0.14* | 0.07 | 0.02 |  |
|  | **ASI** | **BR** | **CC** | **EPP** | **EH** | **EP** | **GY** | **RL** | **SL** | **PH** |

** Significant at P<0.01, *Significant at P<0.05, ASI- Anthesis Silking Interval, BR-Brace Root, CC-Chlorophyll content, EPP-Ears per plant, EH-Ear height, EP-Ear position (EH/PH), GY-Grain Yield, RL-Root Lodging, SL-Stem Lodging and PH-Plant height
